# Massive Bilateral Breast Hypertrophy in Pregnancy Due to Gestational Gigantomastia: A Case Report and Review of the Literature

**DOI:** 10.7759/cureus.113887

**Published:** 2026-08-03

**Authors:** Fatima El Mangoub, Abdelhamid Benlghazi, Bouhtouri Yassine, Mly Mehdi Elhassani, Kouach Jaouad

**Affiliations:** 1 Department of Gynaecology-Obstetrics, Mohammed V Military Teaching Hospital, Rabat, MAR; 2 Department of Obstetrics and Gynaecology, Mohammed V Military University Hospital, Faculty of Medicine and Pharmacy, Mohammed V University, Rabat, MAR

**Keywords:** bilateral breast hypertrophy, bromocriptine, gestational gigantomastia, gigantomastia gravidarum, pregnancy-related complications

## Abstract

Gestational gigantomastia is a very rare disorder characterised by massive, bilateral breast enlargement during pregnancy. Clinically, it is characterised by rapid breast enlargement associated with severe mastodynia, back and shoulder pain, and erythema of the skin. In its most severe forms, it can lead to ulceration, necrosis, haemorrhage, or septicaemia. The underlying pathophysiology is not completely understood, but is thought to be multifactorial, involving hormonal hypersensitivity and autoimmune mechanisms. Histopathological confirmation is necessary for definitive diagnosis because its clinical presentation can closely mimic that of inflammatory breast carcinoma or other malignant neoplasms. We report a case of a 28-year-old primigravida who presented at 24 weeks of gestation with progressive bilateral breast enlargement that reached its maximal extent at 36 weeks and caused significant functional impairment. On clinical examination, there was symmetrical breast hypertrophy with diffuse erythema and a marked peau d'orange appearance. Breast ultrasound revealed glandular hypertrophy with ductal ectasia and marked oedema. No suspicious focal lesions were seen. Hormonal and immunological workup was normal, and skin and glandular biopsy showed benign hyperplasia without atypia. Throughout the pregnancy, conservative management with analgesics and postural support was continued until an uncomplicated vaginal delivery at 39 weeks of gestation. Cabergoline was then administered to suppress lactation. Given the absence of spontaneous regression of the breasts, a multidisciplinary decision was taken to perform a bilateral reduction mammoplasty at 12 months postpartum. This case demonstrates the phenotypic variability of gestational gigantomastia that may present without any preceding breast pathology. In severe cases, surgical treatment (reduction mammoplasty or bilateral mastectomy with delayed reconstruction) is the mainstay of treatment, and bromocriptine is the best-established medical alternative, but with variable success. Coordinated multidisciplinary care is essential to optimise patient outcomes and minimise life-threatening complications.

## Introduction

Gestational gigantomastia is an extremely rare disease that presents with massive breast enlargement during pregnancy. Its incidence is estimated to be one in 28,000 to 100,000 pregnancies [1]. Fewer than 100 cases have been reported in the literature since it was first described in 1684 [2]. The disease usually presents with a rapid bilateral increase in breast volume, associated with back and shoulder pain, skin ulcerations, and even haemorrhages and tissue necrosis, with considerable physical and psychosocial morbidity [3]. The aetiology is unknown, and the clinical presentation may mimic a malignant tumour, so histopathological evaluation is mandatory. Management is still controversial, ranging from conservative medical treatment to breast surgery in severe cases [4]. Given the rarity of this pathology and the lack of data from prospective studies, we report a new clinical case along with a systematic review of the literature with the aim of improving the understanding and management of this rare condition.

## Case presentation

A 28-year-old primigravida, with no significant medical history, presented at 28 weeks of gestation with rapidly progressive bilateral breast enlargement. The initial course of her pregnancy was physiological, with a normal prenatal assessment. The breast enlargement, which began gradually at 24 weeks of gestation, intensified over the following weeks. This hypertrophy was associated with severe and permanent mastodynia in the form of local tension, causing increasing functional impairment.

**Figure 1:**
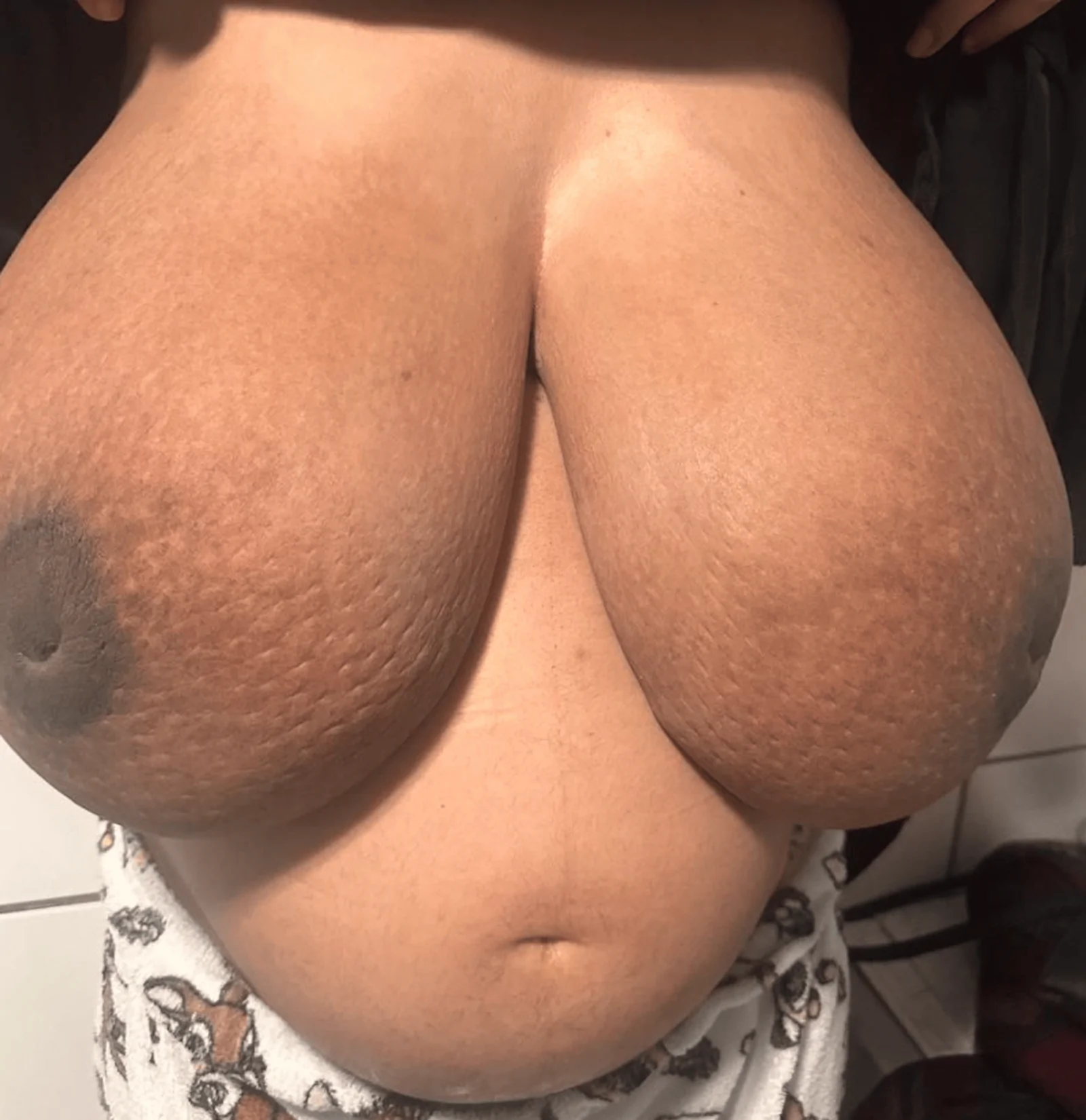
Gigantomastia at 26-week gestational age (frontal view)

On inspection, there was massive, bilateral, symmetrical enlargement of both breasts. The breast skin showed diffuse erythema and a pronounced orange peel appearance, without ulceration or skin necrosis, while a clearly visible superficial venous network was present. On morphological examination, the nipple-sternal fork distance was measured at 34 cm on the right compared to 33 cm on the left. Furthermore, the distance separating the nipple from the base of the breast was 20 cm on the right side and 18.5 cm on the left side (Figure 1).

**Figure 2:**
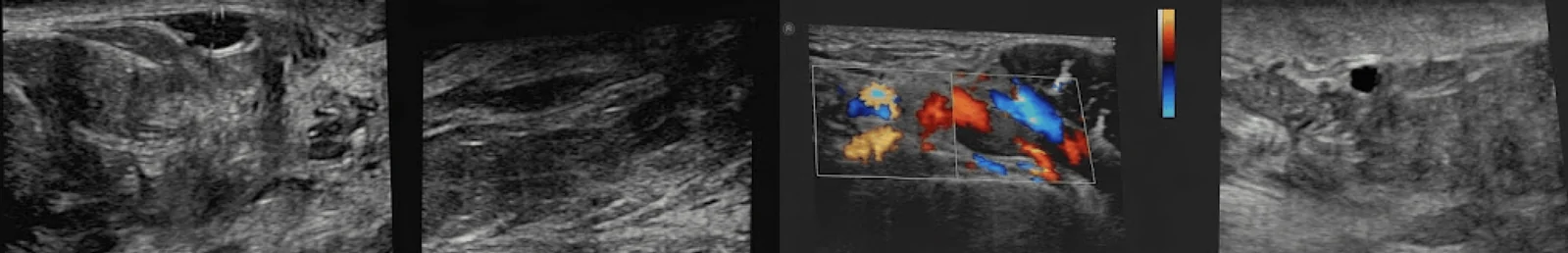
Breast ultrasound Glandular hypertrophy, ductal ectasia, and subcutaneous oedematous changes in the breast noted.

On palpation, the breasts were firm, uniformly tense, and intensely painful, with no identifiable mass or nipple discharge. The axillary lymph node areas were free. Breast ultrasound revealed glandular hypertrophy associated with dilation of the lactiferous ducts and vascular structures. It also revealed significant oedematous infiltration of the skin and subcutaneous tissue, without any underlying suspicious lesion (Figure 2).

In order to formally rule out inflammatory breast carcinoma, a skin-glandular biopsy was performed. Histological analysis showed benign glandular and stromal hyperplasia, without cellular atypia or evidence of malignancy. 

The hormonal profile (prolactin, estradiol, progesterone, testosterone, thyroid-stimulating hormone (TSH), parathyroid hormone) was physiological for the gestational age. The investigations were completed by an immunological assessment (antinuclear, anti-dsDNA, and anti-Ro (SS-A) antibodies), which yielded normal results, excluding an underlying connective tissue disease. Taking into account the intensity of the pain and the functional impact, analgesic treatment adapted to pregnancy was established. First-line treatment was based on paracetamol (1 g every eight hours, with a maximum daily limit of 3 g). Adjuvant physical measures were also implemented: local application of cold compresses, gentle manual lymphatic drainage, and permanent wearing of a custom-made orthopaedic support bra.

**Figure 3:**
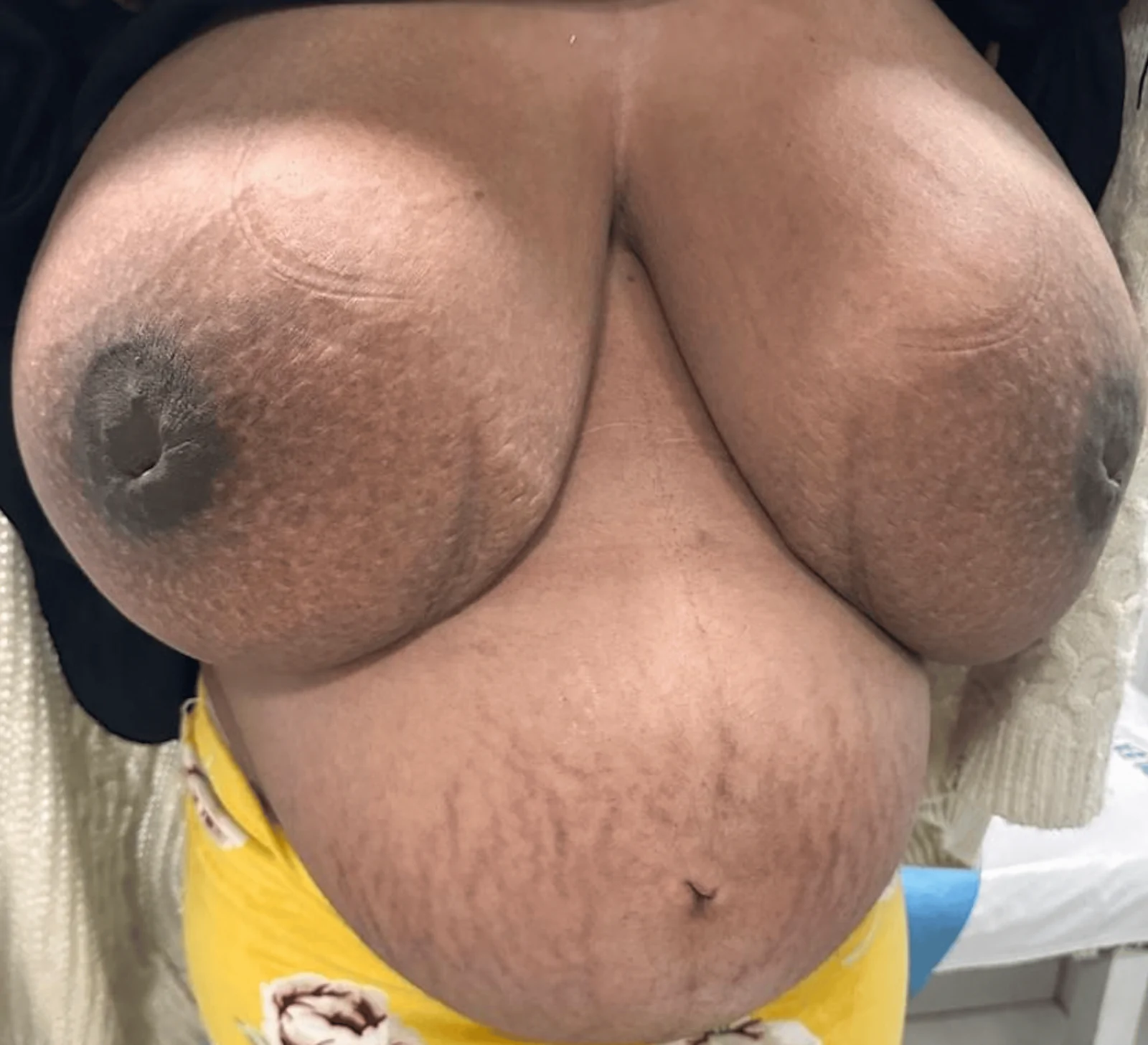
Gigantomastia at 36-week gestational age (frontal view)

At the 32-week follow-up, the increased breast volume was accompanied by significant functional impairment and worsening back pain. At 36 weeks, breast distension reached its maximum with significant skin tension, while erythema and the superficial venous network remained stable (Figure 3).

At 39 weeks, the patient delivered vaginally without complications. A male newborn weighing 3300 g was born with an Apgar score of 10/10 at one and five minutes [5].

Breastfeeding was contraindicated due to the high risk of breast engorgement and ischemic or infectious complications. Lactation suppression was initiated immediately after delivery with a single dose of cabergoline (1 mg). Following delivery, three management options were discussed with the patient: therapeutic abstention with close clinical monitoring, medical treatment with bromocriptine, or breast reduction surgery. By mutual agreement, a conservative strategy based on simple clinical monitoring was chosen. Pain management, including back pain and shoulder pain secondary to breast weight, was treated with paracetamol, enhanced by the additional use of a back support band.

**Figure 4:**
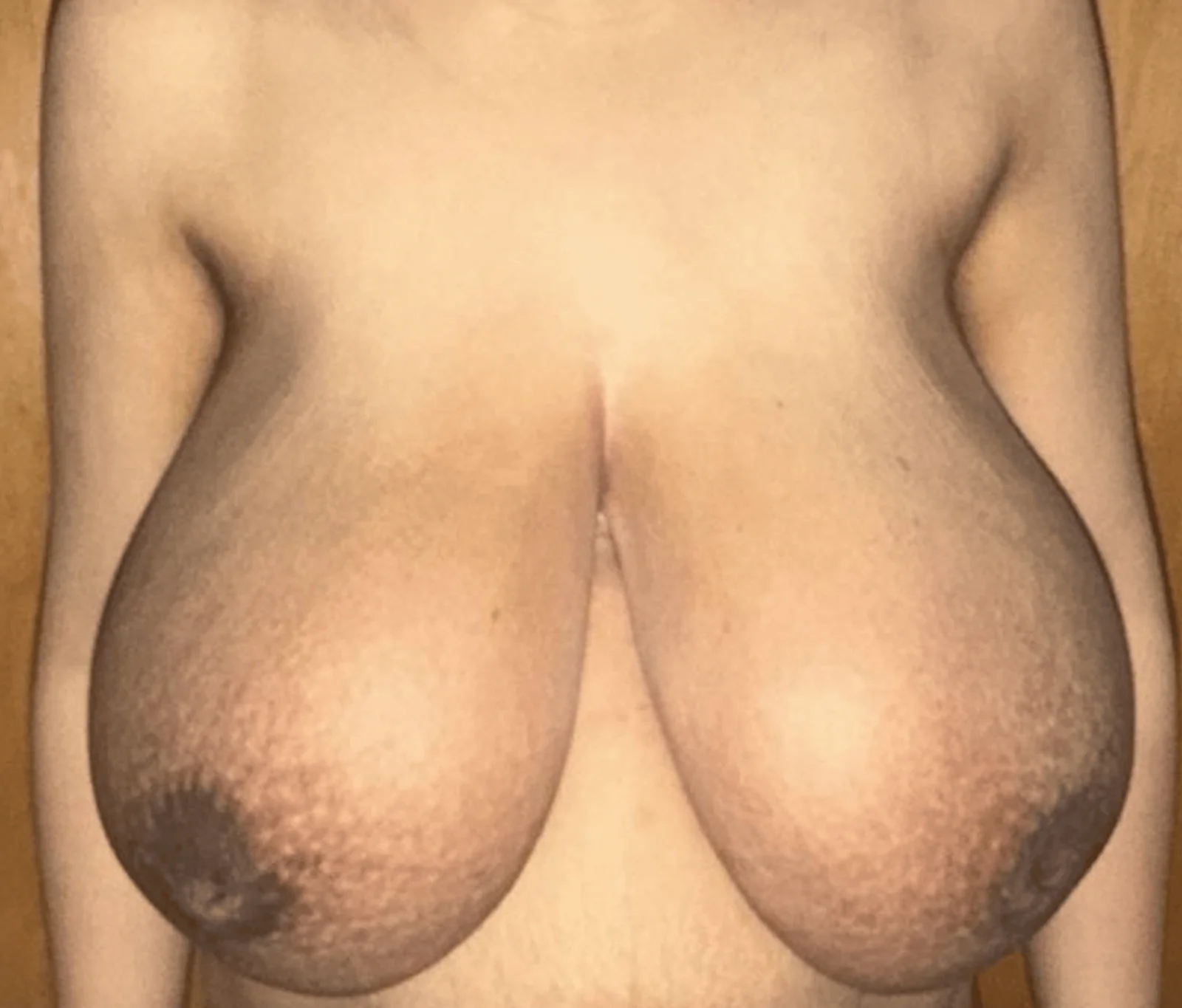
Clinical appearance postpartum

Clinical follow-up revealed persistent hypertrophy. However, the congestive and inflammatory manifestations gradually subsided. The acute congestive pain disappeared, leaving only purely mechanical discomfort related to the weight of the breasts (Figure 4).

After 12 months of regular follow-up, the breast hypertrophy stabilised, confirming the absence of late spontaneous involution. The case was discussed at a multidisciplinary meeting, and bilateral breast reduction for functional and aesthetic purposes was chosen as the definitive treatment option. At the time of writing this report, the procedure is scheduled but has not yet been performed.

## Discussion

Gestational gigantomastia is an extremely rare condition, with an estimated incidence of between one in 28,000 and one in 100,000 pregnancies [1]. A systematic review of the literature in PubMed and Scopus databases for the period 1951-2020 identified 98 cases reported in 84 studies [4]. These results highlight the rarity of the condition and justify the lack of data from large studies.

Gestational gigantomastia predominantly affects young women and tends to manifest early in pregnancy, most frequently during the first trimester [6]. The most common initial symptom is rapid bilateral breast hypertrophy, which occurs in more than 80% of patients [7]. However, the clinical presentation may be atypical: some patients develop their first gestational gigantomastia in late pregnancy, at an advanced maternal age, or without a history of previous breast hypertrophy. These cases illustrate the phenotypic variability of the disease and the need for a high degree of clinical vigilance, regardless of the rank of pregnancy.

The weight of enlarged breasts can reach considerable levels, generating significant mechanical stress on the spine and shoulder girdle. On the skin level, the progressive distension of the integument exposes patients to risks of ulceration, infection, and necrosis, which can lead to potentially fatal systemic complications, including septicemia or haemorrhage [8].

The aetiology of gestational gigantomastia remains uncertain, but the most widely accepted hypothesis involves tissue hypersensitivity or the overproduction of pregnancy-related hormones, particularly prolactin and human chorionic gonadotropin. This is supported by the frequent occurrence of gestational gigantomastia in early pregnancy - a period of hormonal peaks - and the partial efficacy of hormone antagonists in some cases [9]. Nonetheless, gestational gigantomastia can also occur in patients with strictly normal hormonal profiles, which suggests additional mechanisms, particularly autoimmune factors [10]. The concomitant presence of autoimmune diseases in several reports, along with the rarity of abnormal laboratory findings in most cases, further supports a multifactorial pathogenesis [7,11].

Histological analysis of breast tissue generally reveals pregnancy-related changes, including glandular hyperplasia without atypia, connective tissue proliferation, and fibrosis, with no significant inflammatory infiltrate [12]. While these histological features are benign, they can be confused for a tumour, both clinically and on imaging. This similarity is particularly relevant because certain malignant tumours - such as invasive ductal carcinoma, lobular carcinoma in situ, and lymphomas - can cause a massive increase in breast volume and mimic giant cell tumours [13]. Furthermore, other differential diagnoses must also be considered, such as phyllodes tumours and giant fibroadenomas, which most often present unilaterally and are unrelated to pregnancy. Consequently, a rigorous histopathological evaluation is essential to establish a definitive diagnosis [14].

The management of gestational gigantomastia relies on an individualised approach, taking into account disease severity, the stage of pregnancy, and the patient's desires. Medical treatment with hormone antagonists (bromocriptine) is the first-line therapeutic approach [15]. Prescribed in 54.28% of cases reported in the literature, bromocriptine aims to counteract the hormonal stimulation [16]. However, its effectiveness is variable and most often transient; while it can halt progression or induce partial regression, it generally does not restore initial breast volume. Other pharmacological agents have been used, including progesterone, tamoxifen, and danazol, with similarly inconsistent results [17]. Table 1 summarises the reported cases of gestational gigantomastia published between 2016 and 2026, highlighting patient characteristics, clinical presentation, and management modalities.

**Table 1:** Summary of gestational gigantomastia cases and their management modalities reported in the literature (2016-2026) This table summarises the reported cases of gestational gigantomastia published between 2016 and 2026, highlighting patient characteristics, clinical presentation, and management modalities, including medical treatment, surgical intervention, and pregnancy outcomes. ADM: Acellular dermal matrix; ANA: Antinuclear antibodies; DIEP: Deep inferior epigastric artery perforator (flap); ER: Estrogen receptor; FSH: Follicle-stimulating hormone; GxPy: Obstetric history notation where G (gravida) represents the total number of pregnancies and P (para) represents the number of viable births/deliveries; LD: Latissimus dorsi; NEC: Necrotising enterocolitis; NICU: Neonatal intensive care unit; NR: Not reported/normal range; PASH: Pseudoangiomatous stromal hyperplasia; PR: Progesterone receptor; PTH: Parathyroid hormone; PTHrP: Parathyroid hormone-related protein; RDS: Respiratory distress syndrome; TSH: Thyroid-stimulating hormone; wks: Weeks; 1,25(OH)_2_D: 1,25-dihydroxyvitamin D (calcitriol)

Author(s)	Year	Country	Age	Parity	Gestational age at diagnosis	Clinical presentation	Suspected/associated aetiology	Hormonal/laboratory workup	Medical treatment	Time from diagnosis to surgery	Surgical treatment	Excised breast tissue weight (g/kg)	Type of nipple grafting	Maternal/fetal outcome	Recurrence
Gerall and Jatoi [3]	2019	USA	31	G5P4	18	Rapid bilateral breast enlargement, erythema, pain	Idiopathic	Endocrine workup normal	Bromocriptine	~4 months (postpartum)	Bilateral reduction mammoplasty (4 months postpartum)	NR	NR	Healthy baby	NR
Rakislova et al. [4]	2020	Mozambique	30	G2P1	24	Rapid bilateral breast enlargement, pain, discomfort	Idiopathic	HIV+, limited workup	Antibiotics (penicillin G)	Surgery declined/not performed	No surgery performed	NR	NR	Maternal death (hypovolemic shock, day 13), twin pregnancy	NR
Qin et al. [6]	2020	China	25	G1P1	Onset during pregnancy	Rapid bilateral breast enlargement, pain, discomfort	Hormonal	Elevated prolactin	Bromocriptine	Postpartum (timing not specified)	Bilateral mastectomy + tissue expanders, later replaced with definitive implants at 8 months	Right: 3975 g, Left: 4728 g	Free nipple graft	Healthy baby, good cosmetic outcome	No (1-year follow-up)
Almajed et al. [7]	2025	Saudi Arabia	36	G4P3	Onset 2nd trimester (4th pregnancy), presented 6 months postpartum	Rapid bilateral breast enlargement, severe back pain, shoulder numbness.	Hormonal	Prolactin and progesterone elevated	None	1 month after presentation	Bilateral reduction mammoplasty + free nipple graft	Right: 5.150 kg / Left: 5.530 kg (total 10.68 kg)	Free nipple graft	Good recovery, resolution of musculoskeletal symptoms	NR
Abdulkarim et al. [8]	2024	Canada	29	G1P0	23	Rapid bilateral breast enlargement, back pain, discomfort, dyspnea	Idiopathic	Endocrine workup normal	None	Immediate (2nd trimester)	Bilateral skin - sparing mastectomy + immediate reconstruction (Goldilocks technique) + free nipple grafting	~7 kg/breast (≈14 kg total)	Free nipple graft	Full-term vaginal delivery, good outcome	NR
Modarressi et al. [9]	2018	USA	33	G8P4	8	Rapid bilateral breast enlargement, pain	Hormonal	Elevated PTHrP, suppressed PTH, 1,25(OH)_2_D	IV saline, prednisone, calcitonin, cinacalcet (off-label)	Termination at 20 wks, mastectomy 6 months later	Elective termination of pregnancy (20 wks), then bilateral subcutaneous mastectomy 6 months later	NR	NR	Rapid resolution of hypercalcemia after termination (without mastectomy)	NR
Young et al. [10]	2023	USA	23	G1P0	19	Erythema, pruritus, peau d'orange, stellate plaques (necrosis), induration	Autoimmune (lupus mastitis)	ANA 1:2560 (speckled), anti-Ro+, prolactin normal	Cabergoline + hydroxychloroquine	1 year (postpartum reduction)	Reduction mammoplasty (1 year postpartum)	NR	NR	Uneventful delivery, improvement of skin lesions	NR
Mangla et al. [11]	2019	India	21	G1P0	24	Massive bilateral enlargement, severe back pain	Autoimmune (myasthenia gravis)	Prolactin mildly elevated, TSH: low	Cabergoline	Reduction mammoplasty planned, not performed at time of publication	Reduction mammoplasty planned (not yet performed)	Estimated pre-treatment weight: 6 kg (R) / 7 kg (L)	NR	Full-term vaginal delivery, healthy infant 3.2 kg	NR
Diallo and Ba [12]	2019	Senegal	29	G2P2	28	Bilateral enlargement impairing daily activities, bilateral breast ulceration	Hormonal	Markedly elevated prolactin, normal FSH/LH	Bromocriptine	No surgery (conservative follow-up)	No surgical treatment	NR	NR	Decrease in size and regression of ulcerations 6 months postpartum (vaginal delivery)	NR
Fletcher et al. [13]	2020	USA	31	G5P4	18	Bilateral swelling, erythema, pain, spontaneous local haemorrhage (left breast)	Idiopathic	ANA low positive (1:40), prolactin/estradiol/progesterone/TSH normal	Bromocriptine	Postpartum (timing not specified)	Postpartum reduction mammoplasty	NR	NR	Term cesarean, healthy infant; went from M-cup to D-cup with relief	NR
Kaluarachchi et al. [14]	2018	Sri Lanka	34	G3P2	20	Progressive diffuse bilateral enlargement	Idiopathic	Hormone profile and thrombophilia screen normal	Bromocriptine + low molecular weight heparin	Mastectomy at 24 wks (4 wks after presentation)	Bilateral total mastectomy including removal of axillary tails at 24 wks	Right: breast 5.5 kg + axillary tail 2.2 kg / Left: breast 6.6 kg + axillary tail 2.1 kg	None (simple mastectomy, no reconstruction)	Vaginal delivery at 37 wks, infant 2.6 kg	NR
Dharini et al. [16]	2018	India	20	G1P0	24	Rapidly increasing size of both breasts, upper back pain	Idiopathic	Elevated: estradiol, progesterone, prolactin	Observation only	No surgery performed	No surgical treatment (spontaneous resolution)	NR	NR	Termination at 28 wks (pre-eclampsia), neonatal death on day 3; spontaneous resolution of breast volume over 7 months	NR
Mahabbat et al. [17]	2020	Saudia Arabia	33	G3P2	23	Breast enlargement, skin ulceration, discharge	Idiopathic	PASH on histology, CD34+, ER/PR negative	Bromocriptine, IV antibiotics (amoxicillin/clavulanate, meropenem)	A few days (rapid worsening)	Bilateral skin-sparing mastectomy + immediate reconstruction with pre-pectoral implant/ADM, multiple revisions (infection, implant removal), then bilateral LD flaps	Right: 5.08 kg / Left: 4.49 kg	Free graft (delayed NAC reconstruction)	Vaginal delivery at 39 wks, infant 2.58 kg	NR
Hassayoune et al. [18]	2021	Belgium	29	NR	15	Breast enlargement, dyspnea, chest tightness, recurrent haemorrhage, multiple ulcerations	Idiopathic	Prolactin normal inflammatory syndrome	Bromocriptine	Emergency postpartum mastectomy (after cesarean at 32 wks)	Emergency bilateral skin-sparing mastectomy postpartum, NAC banking, then delayed reconstruction with tissue expanders followed by bilateral DIEP flaps + nipple grafting	Right: 9.315 kg / Left: 6.430 kg	Delayed free graft (banked then grafted onto DIEP flaps)	Cesarean at 32 wks, healthy infant; maternal *Pseudomonas *sepsis postpartum	NR
Lemma et al. [19]	2025	Ethiopia	26	G1P0	8	Excessive breast enlargement	Idiopathic	-	None	~3 years from onset (resource-limited setting decision)	Bilateral simple mastectomy + NAC reconstruction with full-thickness skin graft	NR (weight not specified)	Free graft (full-thickness skin graft)	Neonatal death; good maternal recovery, patient satisfied	NR
Neblett et al. [20]	2021	Jamaica	34	G4P2	22	Rapid bilateral breast enlargement, chest and back pain	Hormonal	Mildly elevated estradiol and prolactin	Bromocriptine	Emergency (postpartum day 4), then 2 wks later (contralateral)	Right total mastectomy, followed by elective left total mastectomy 2 wks later	Right: 2940 g / Left: 4750 g	None (simple mastectomy, no reconstruction)	Cesarean at 33 wks, healthy infant 1.55 kg	Delayed reconstruction declined
Pedreira and Santiago [21]	2017	Brazil	33	NR	15	Massive bilateral enlargement, local pain and hyperemia	Autoimmune (systemic lupus erythematosus)	Hormones normal, ANA 1:160 (pre-existing)	Hydroxychloroquine	Mastectomy at 26 wks (during pregnancy)	Bilateral radical mastectomy at 26 wks	7.5 kg/breast (15 kg total)	NR	No reported neonatal complications	-
Cabrera et al. [22]	2020	USA	31	G1P0	30	Increase in breast size, pain	Hormonal	PTH, PTHrP, vitamin D, prolactin, TSH: normal	Bromocriptine, then cabergoline, then high-dose bromocriptine; manual lymphatic drainage, compression garment	20 wks postpartum	Bilateral reduction mammoplasty + free nipple graft at 20 wks postpartum	Left: 4.320 kg / Right: 4.762 kg	Free nipple graft	Cesarean at 31 wks, NICU 6 wks (RDS, stage II NEC)	NR
Zingaretti et al. [23]	2017	Italy	37	G2P1	23	Bilateral breast swelling, peau d'orange, ulceration, necrosis	Autoimmune (undifferentiated connective tissue disease)	ANA 1:2560, elevated anti-dsDNA, hormones normal	Antibiotics, daily dressings, analgesics	6 wks postpartum	Bilateral reduction mammoplasty + free nipple graft at 6 wks postpartum	Right: 3.23 kg / Left: 4.20 kg	Free nipple graft	Cesarean at 38 wks, concurrent sterilisation; no recurrence at 12 months	No (12-month follow-up)

When medical treatment is insufficient, or in patients with complications, surgery becomes necessary. Two surgical approaches are currently used: reduction mammoplasty and bilateral mastectomy with delayed reconstruction. Breast reduction offers the advantages of a single-stage procedure and the potential preservation of breastfeeding; however, it is associated with a 100% recurrence rate in subsequent pregnancies due to the persistence of breast tissue responsive to hormonal stimulation [18]. Bilateral mastectomy with delayed reconstruction, although more invasive, minimises the risk of recurrence and is associated with less blood loss. It represents the standard treatment option for women planning a future pregnancy [19].

When surgery is required during pregnancy, it should ideally be postponed until fetal viability is attained [20]. The administration of corticosteroids to promote fetal lung maturation in cases of anticipated preterm delivery must be systematically considered [21]. A multidisciplinary approach involving obstetricians, neonatologists, endocrinologists, and psychologists is essential to optimise maternal and fetal outcomes [22].

Since gestational gigantomastia is a progressive condition, the maternal prognosis is closely linked to early diagnosis and the rapid initiation of treatment. Although gestational gigantomastia is a benign histological condition, it can lead to serious and potentially fatal maternal complications and carries a mortality rate of approximately 2% [22]. Regular prenatal consultations are critical to ensure early detection and management of the disease before the onset of potentially irreversible complications [23]. Furthermore, strengthening public health infrastructure and improving the quality of obstetric care are essential levers to reduce maternal morbidity and mortality associated with this disease [23].

## Conclusions

Gestational gigantomastia is a rare but potentially serious condition, the prognosis of which is closely linked to early diagnosis and the quality of care. While most patients recover well after appropriate management, severe or complicated cases can be life-threatening. Although medical treatment is the first-line approach, reduction mammoplasty or mastectomy is often necessary. A multidisciplinary approach and shared decision-making with the patient are essential to optimise both functional and aesthetic outcomes.
